# Supplementary material for: Identification of Cardiovascular Disease-Related Genes Based on the Co-Expression Network Analysis of Genome-Wide Blood Transcriptome
Source: Cells. 2022 Sep 14;11(18):2867. doi: 10.3390/cells11182867 (PMC9496853; doi:10.3390/cells11182867)
Supplement: Supplementary file 1 [file cells-11-02867-s001.zip › cells-1847709-supplementary.pdf]

Identification of Cardiovascular Disease-related Genes Based on the Co-expression Network Analysis of Genome-wide Blood Transcriptome (Supplementary Materials)

Taesic Lee[1, 2†], Sangwon Hwang[3†], Dong Min Seo[4], Ha Chul Shin[5], Hyun Soo Kim[5], Jang-Young Kim[6] and Young Uh[7*]

[1] Division of Data Mining and Computational Biology, Institute of Global Health Care and Development, Wonju Severance Christian Hospital, Wonju 26411, Korea
[2] Department of Family Medicine, Yonsei University Wonju College of Medicine, Wonju, Korea
[3] Artificial Intelligence Bigdata Medical Center, Yonsei University Wonju College of Medicine, Wonju, Korea
[4] Department of Medical Information, Yonsei University Wonju College of Medicine, Wonju, Korea
[5] Pharmicell Co, Ltd, Seongnam, Korea
[6] Department of Internal Medicine, Yonsei University Wonju College of Medicine, Wonju, Korea
[7] Department of Laboratory Medicine, Yonsei University Wonju College of Medicine, Wonju, Korea
† These authors have contributed equally to this work.

*** Correspondence:**
Young Uh
u931018@yonsei.ac.kr

Supplementary Figure S1. Module selection based on the differential expression analysis

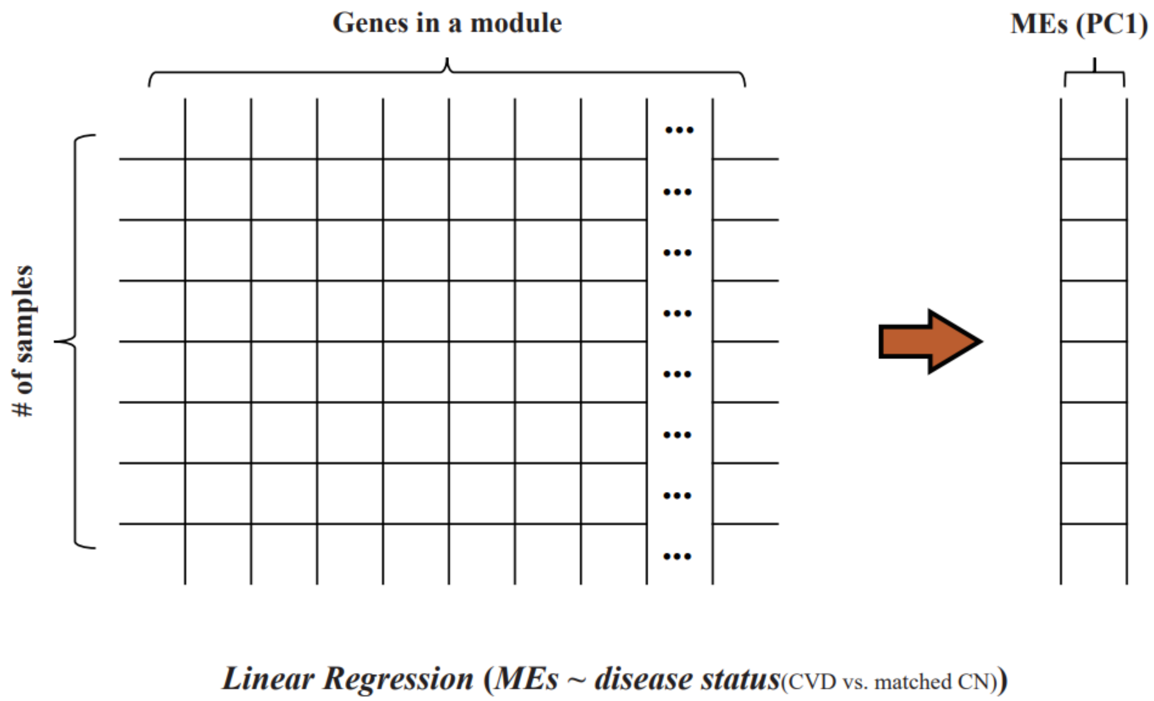

Supplementary Figure S2. Batch normalization via ComBat method.

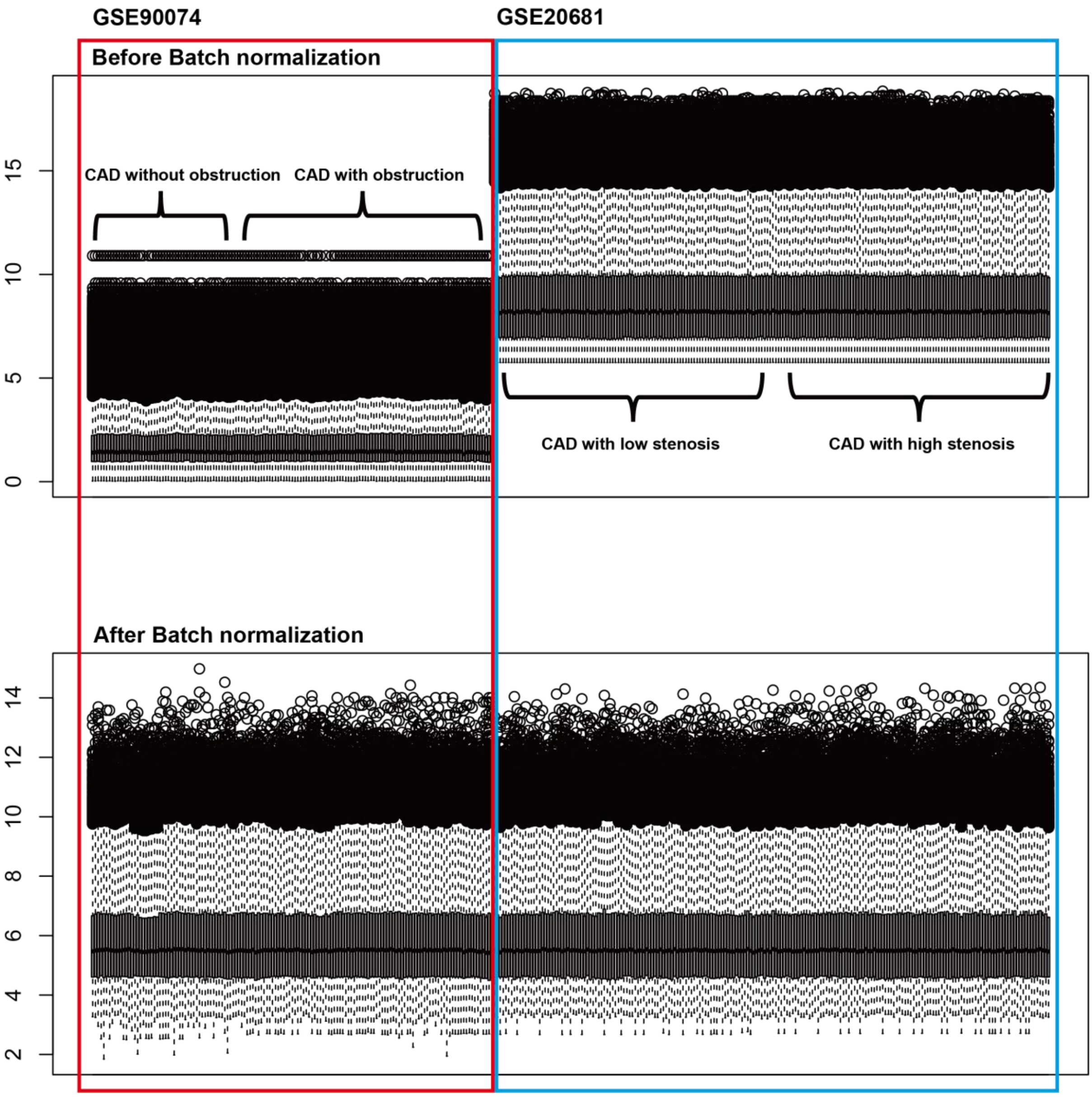

Supplementary Figure S2. Batch normalization via ComBat method (continued).

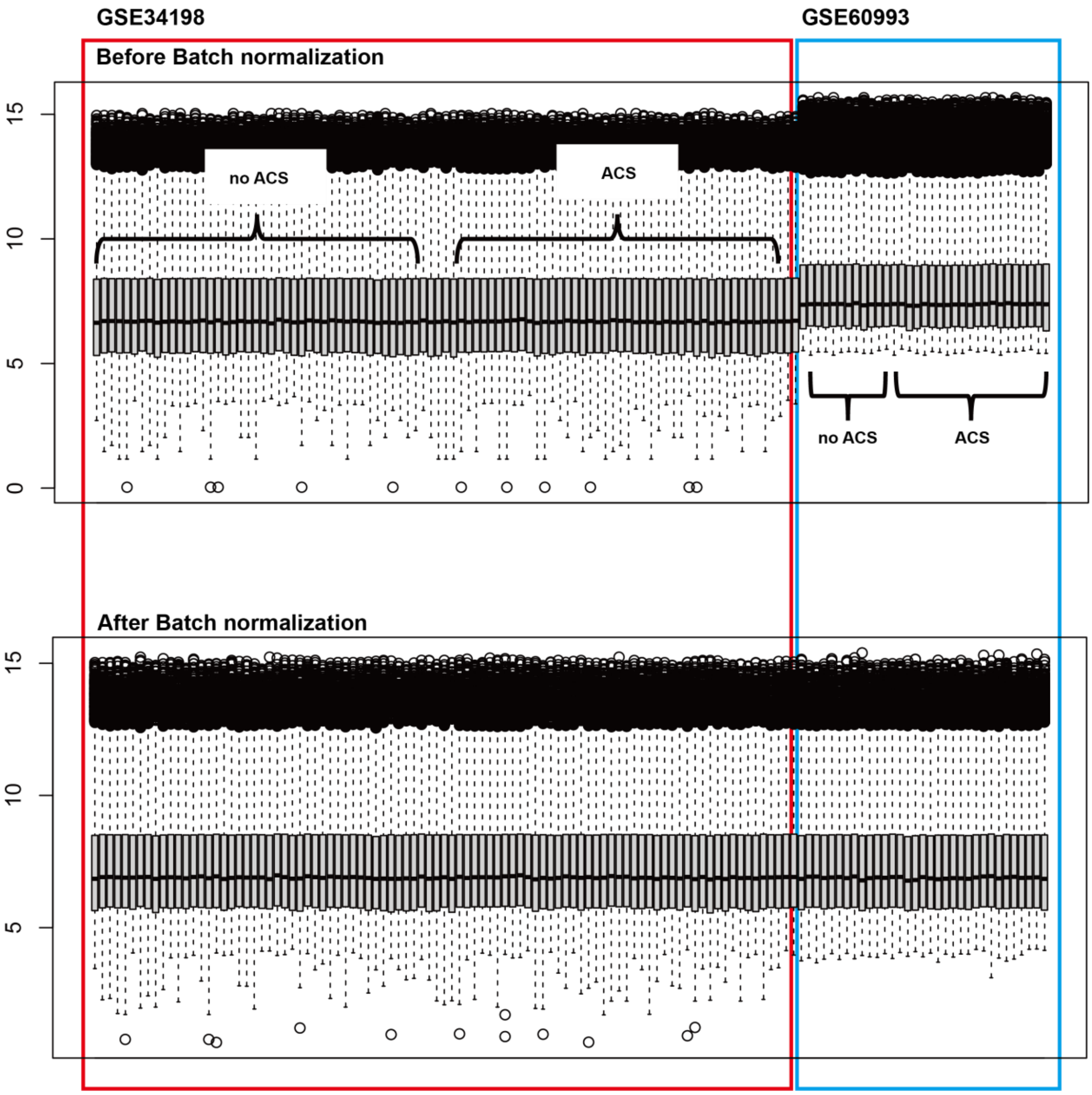

Supplementary Figure S3. Selection of soft threshold power for the establishment of adjacency matrix.

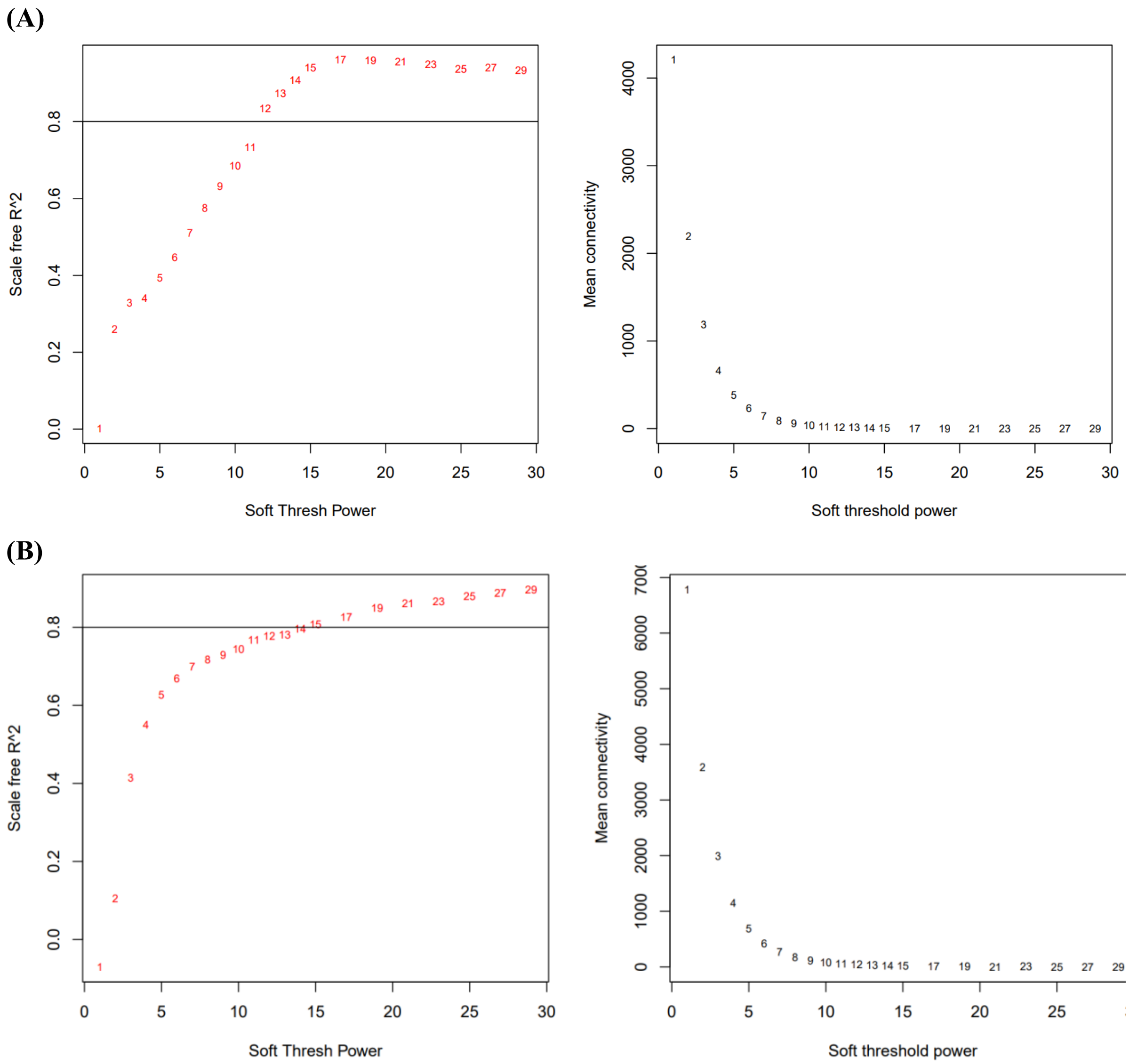

Supplementary Figure S4. Selection of parameters for the construction of module (CAD)

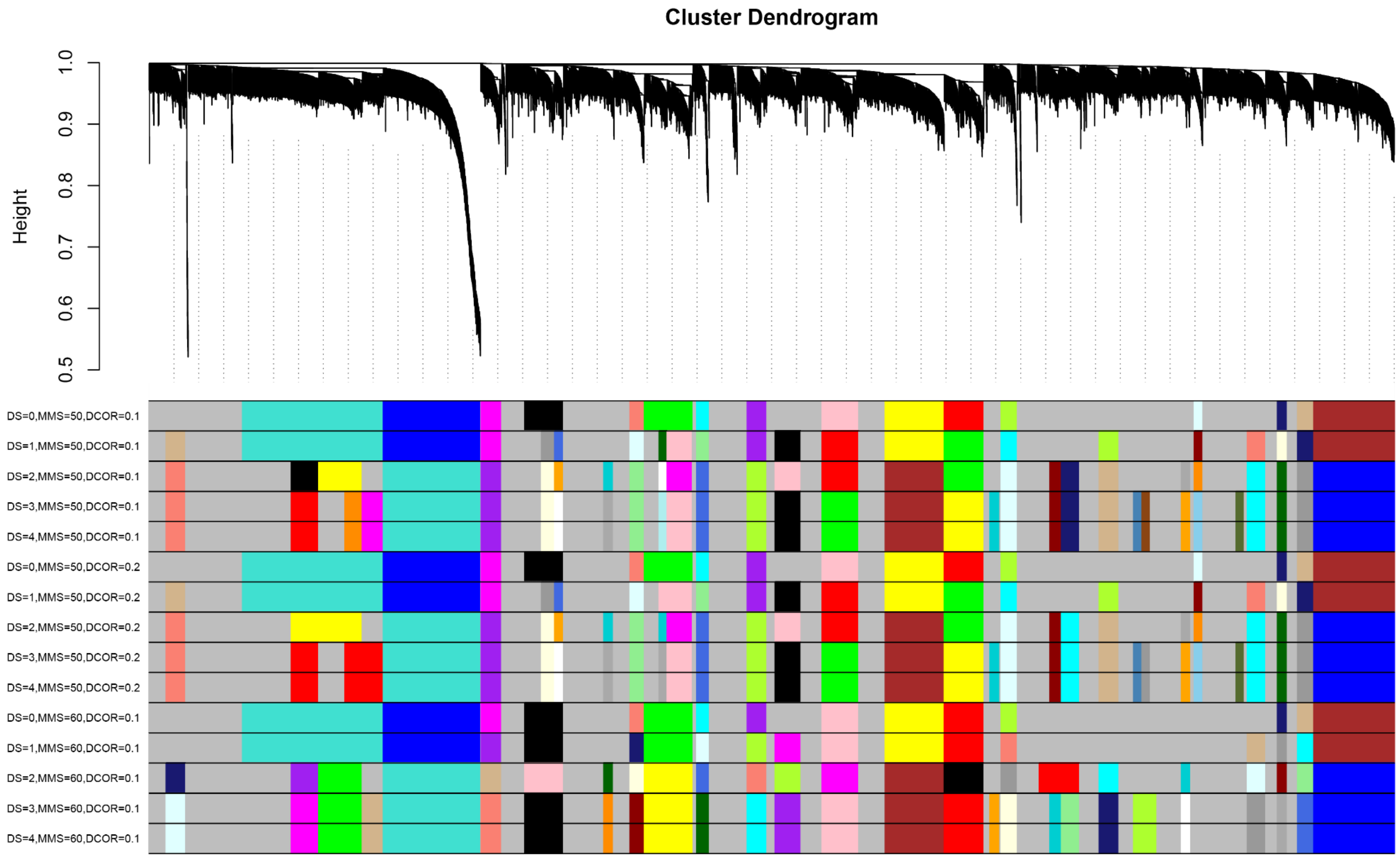

Supplementary Figure S4. Selection of parameters for the construction of module (CAD, Continued)

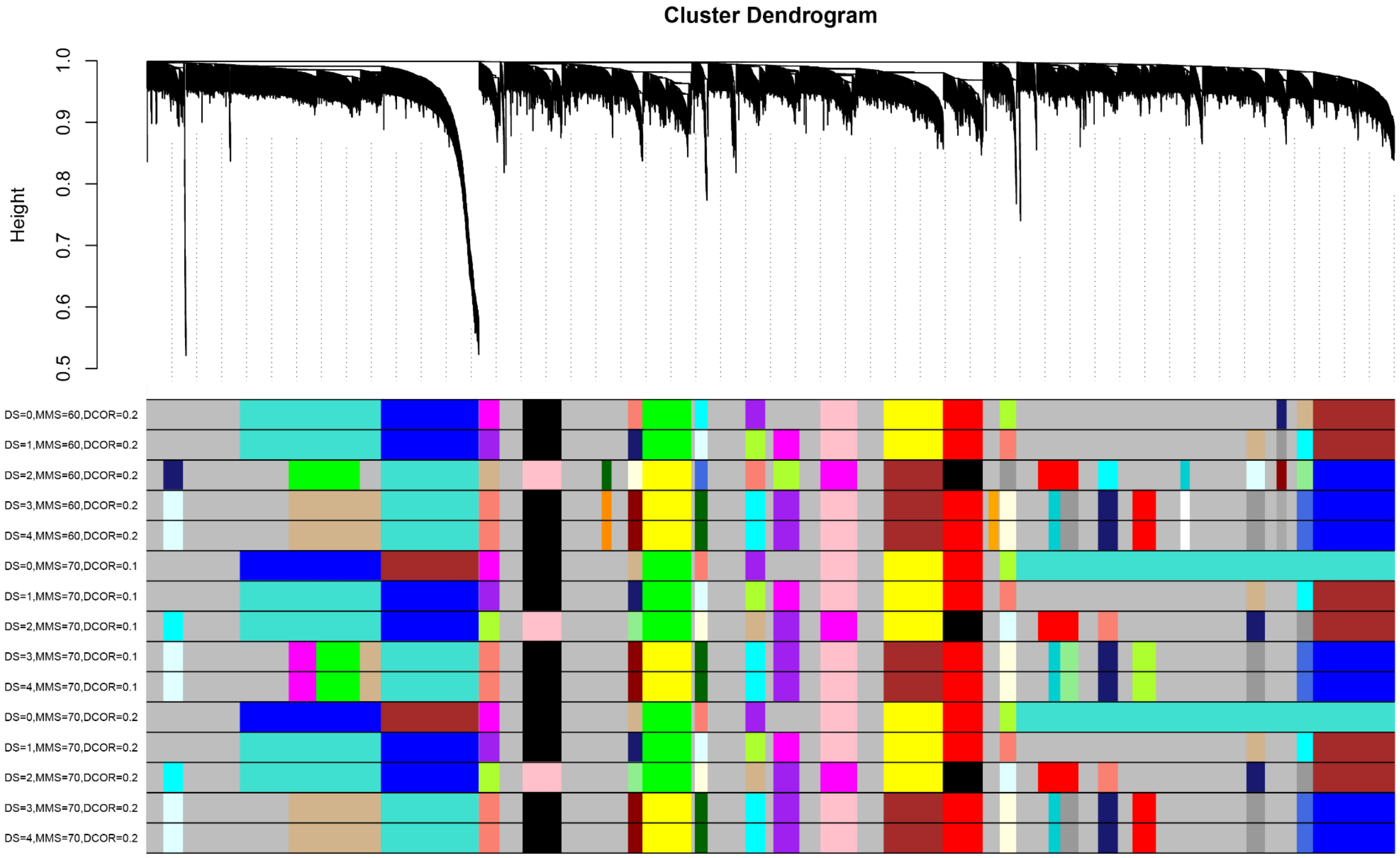

Supplementary Figure S4. Selection of parameters for the construction of module (CAD, Continued)

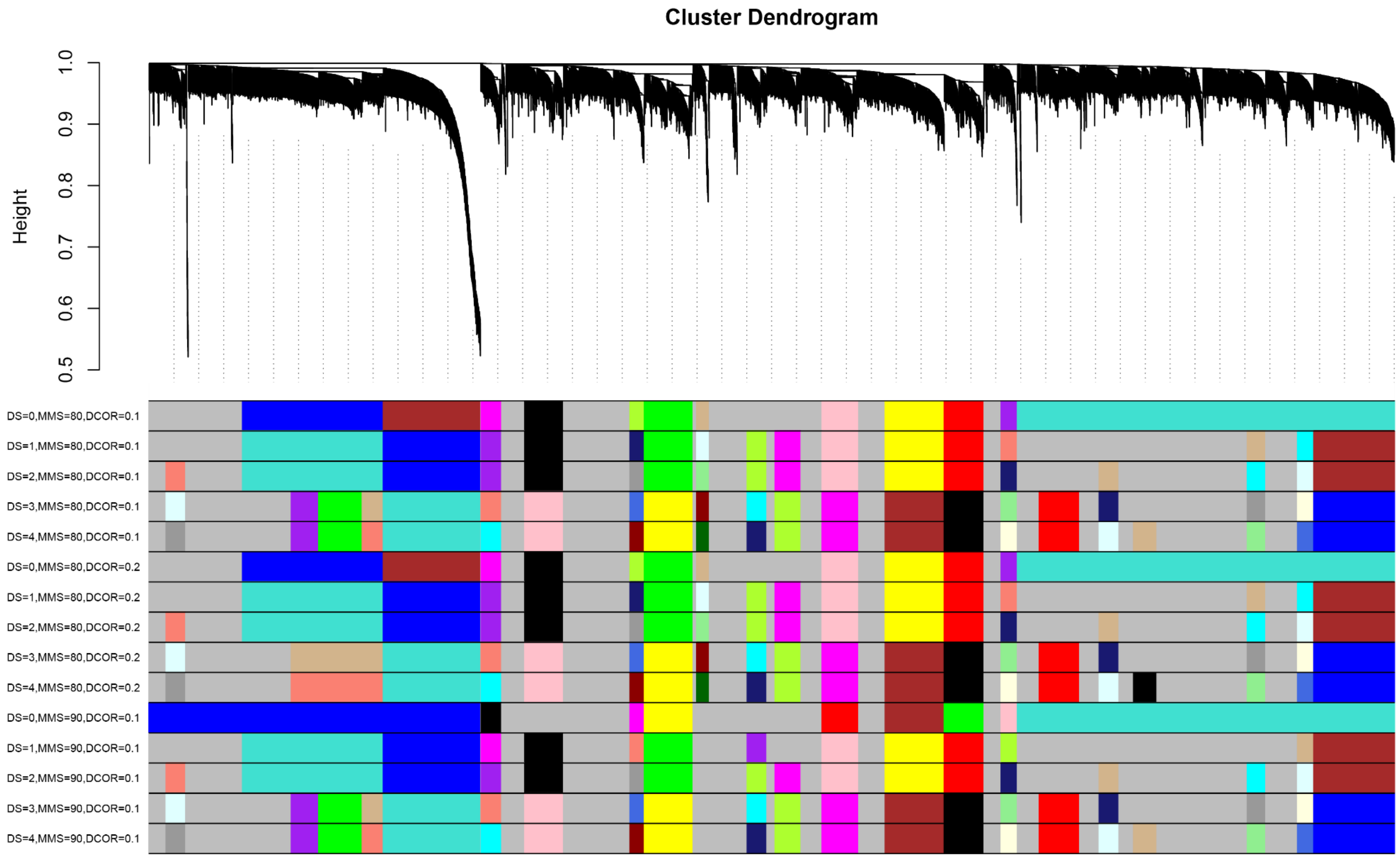

Supplementary Figure S4. Selection of parameters for the construction of module (CAD, Continued)

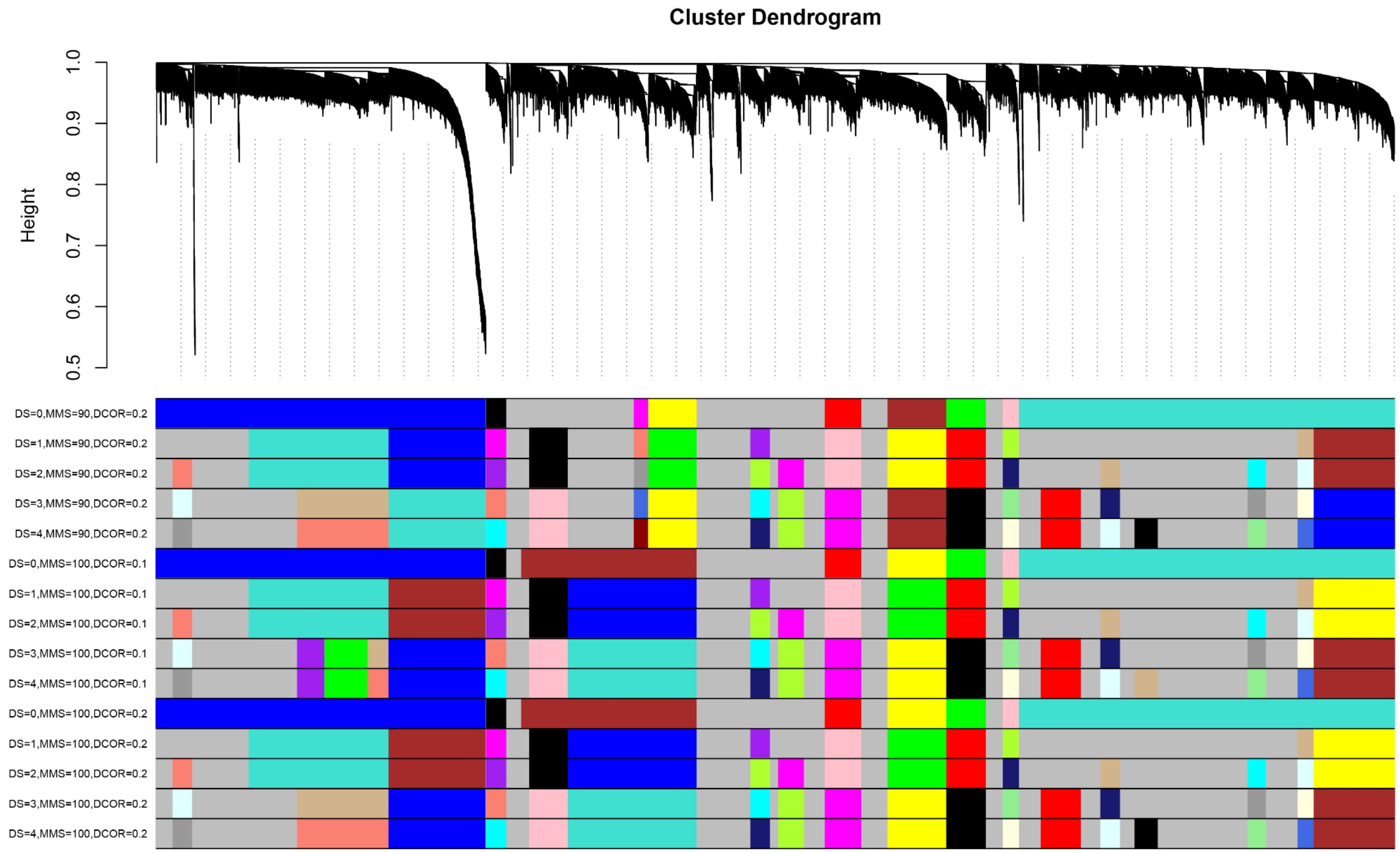

Supplementary Figure S5. Selection of parameters for the construction of module (ACS)

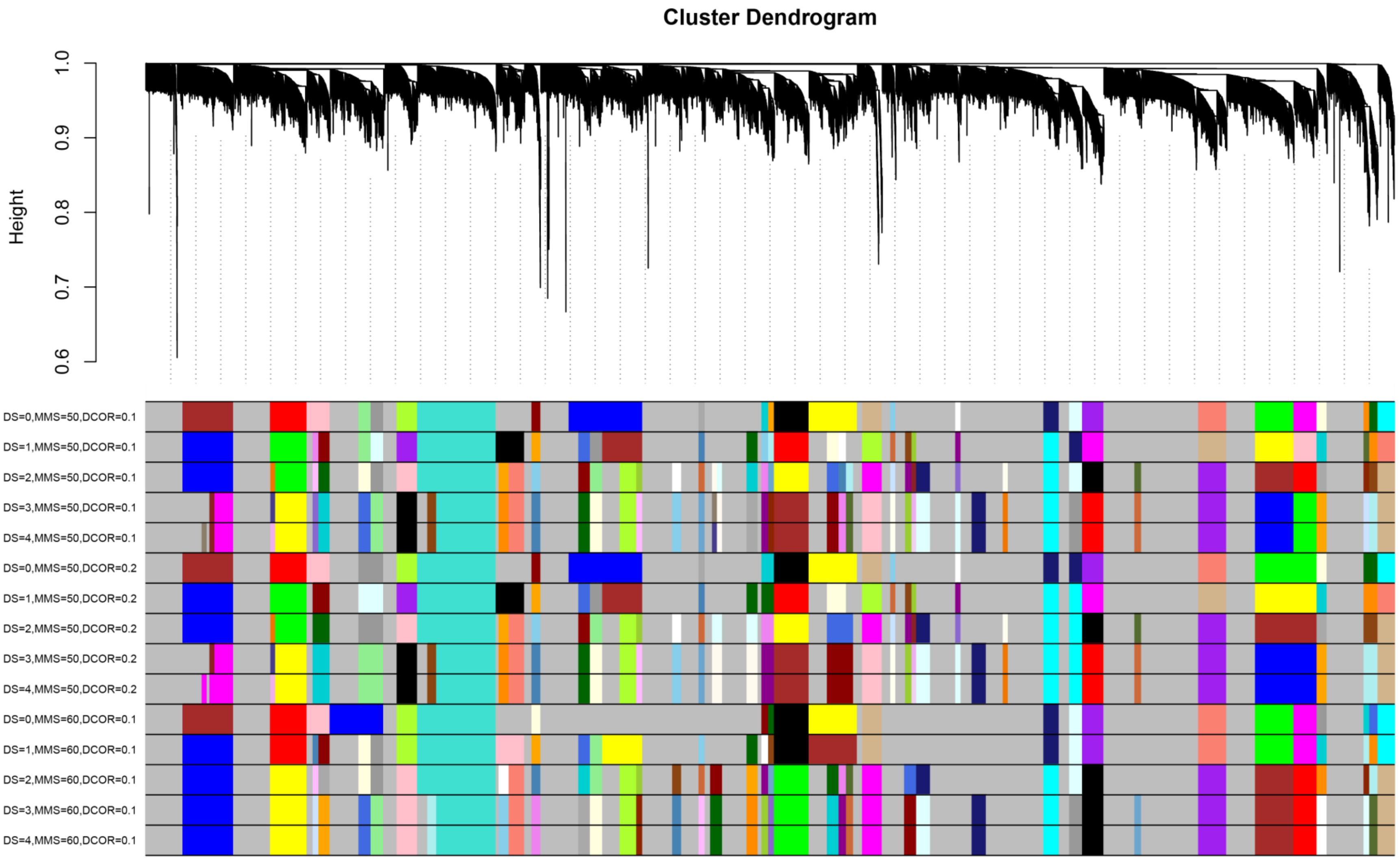

Supplementary Figure S5. Selection of parameters for the construction of module (ACS, Continued)

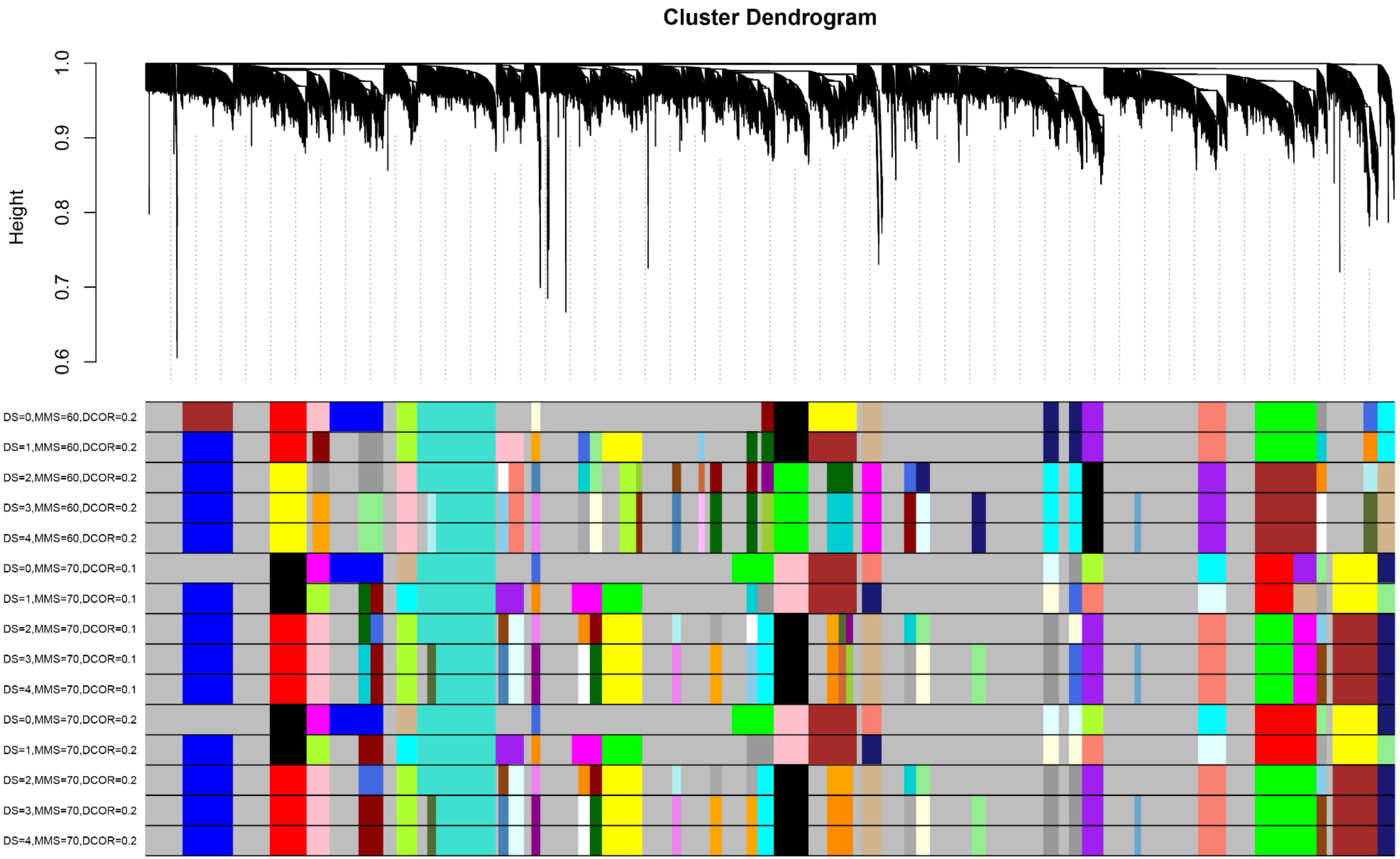

Supplementary Figure S5. Selection of parameters for the construction of module (ACS, Continued)

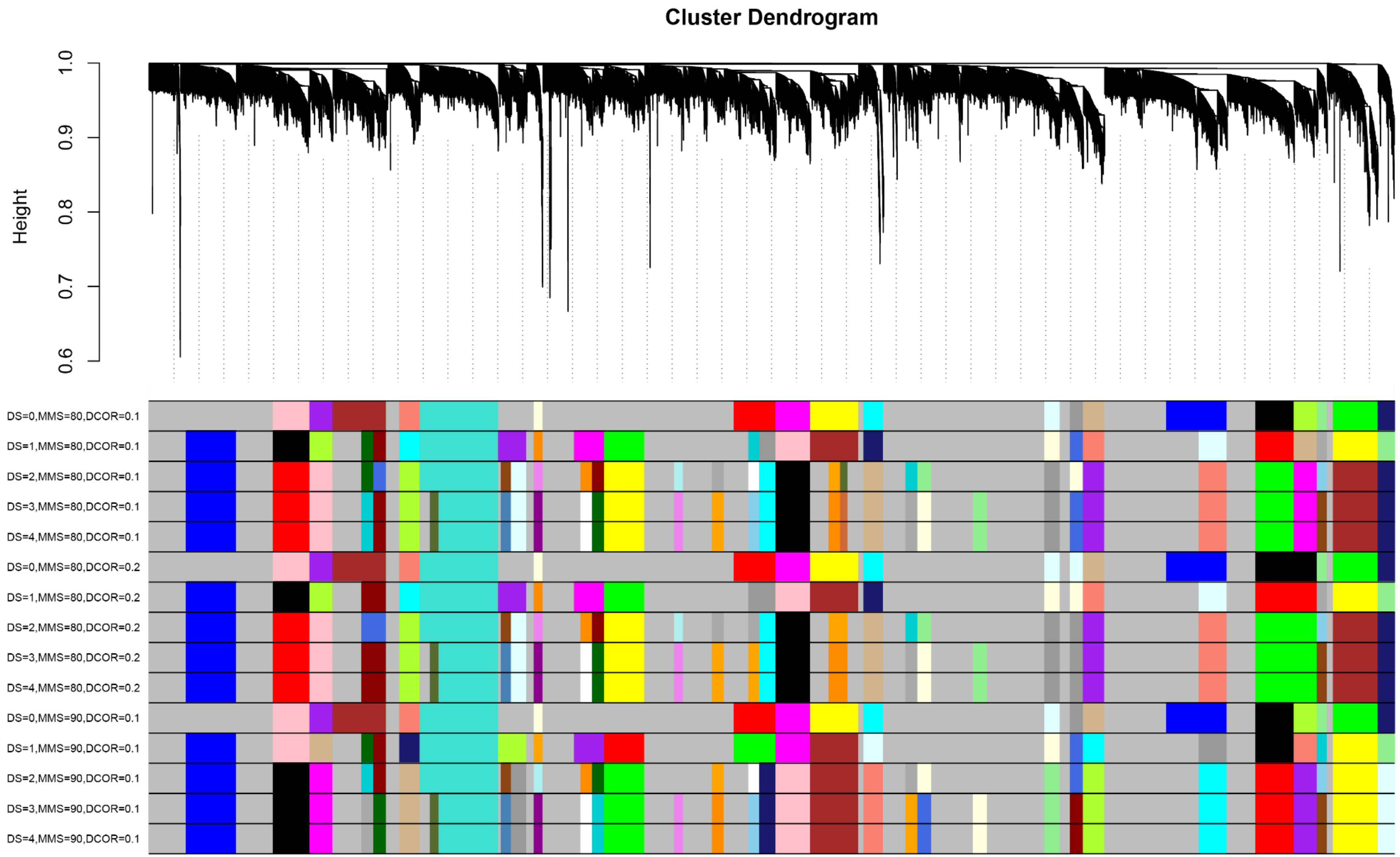

Supplementary Figure S5. Selection of parameters for the construction of module (ACS, Continued)

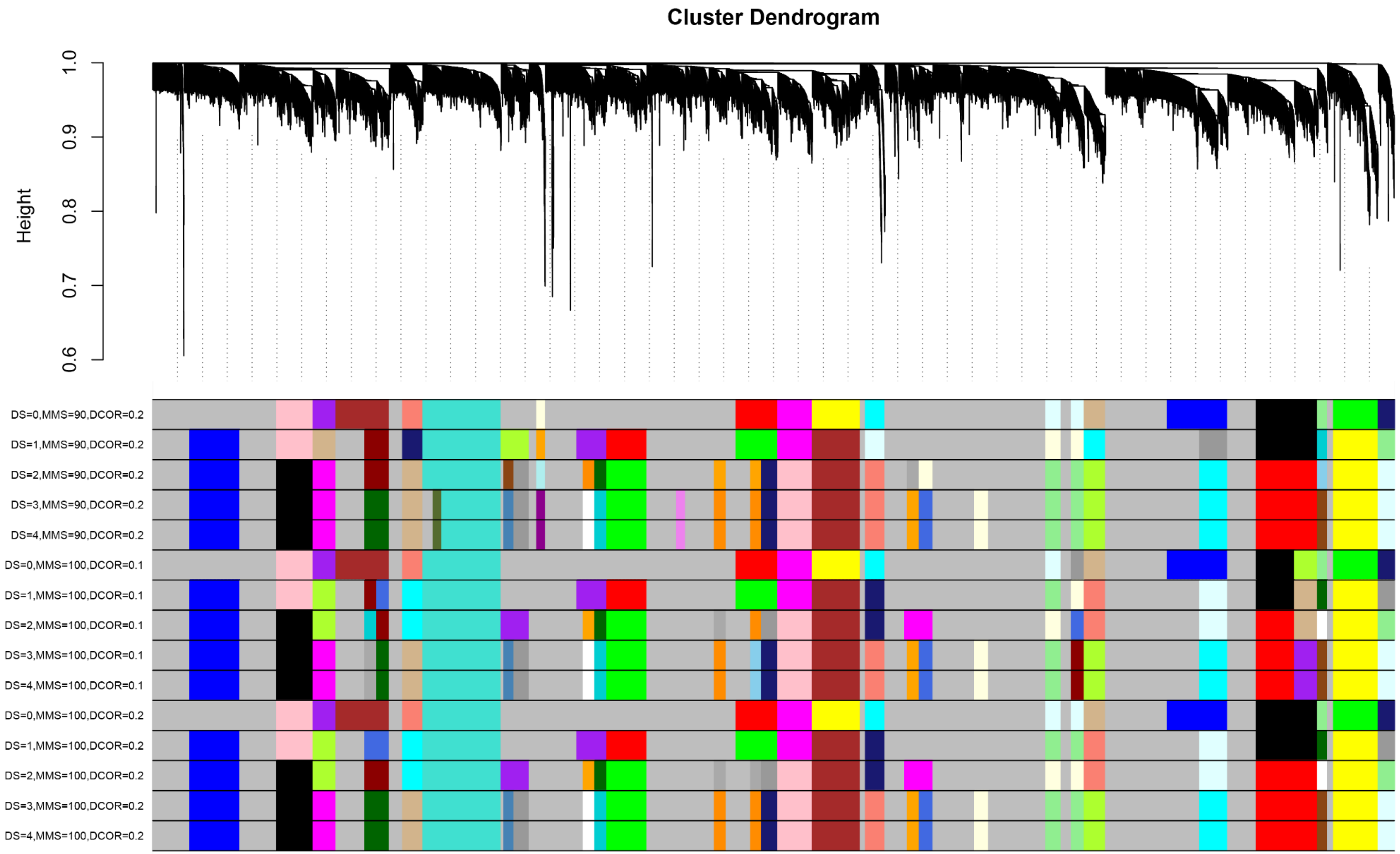

Supplementary Figure S6. Obstructive CAD-related modules selection based on the differential expression analysis.

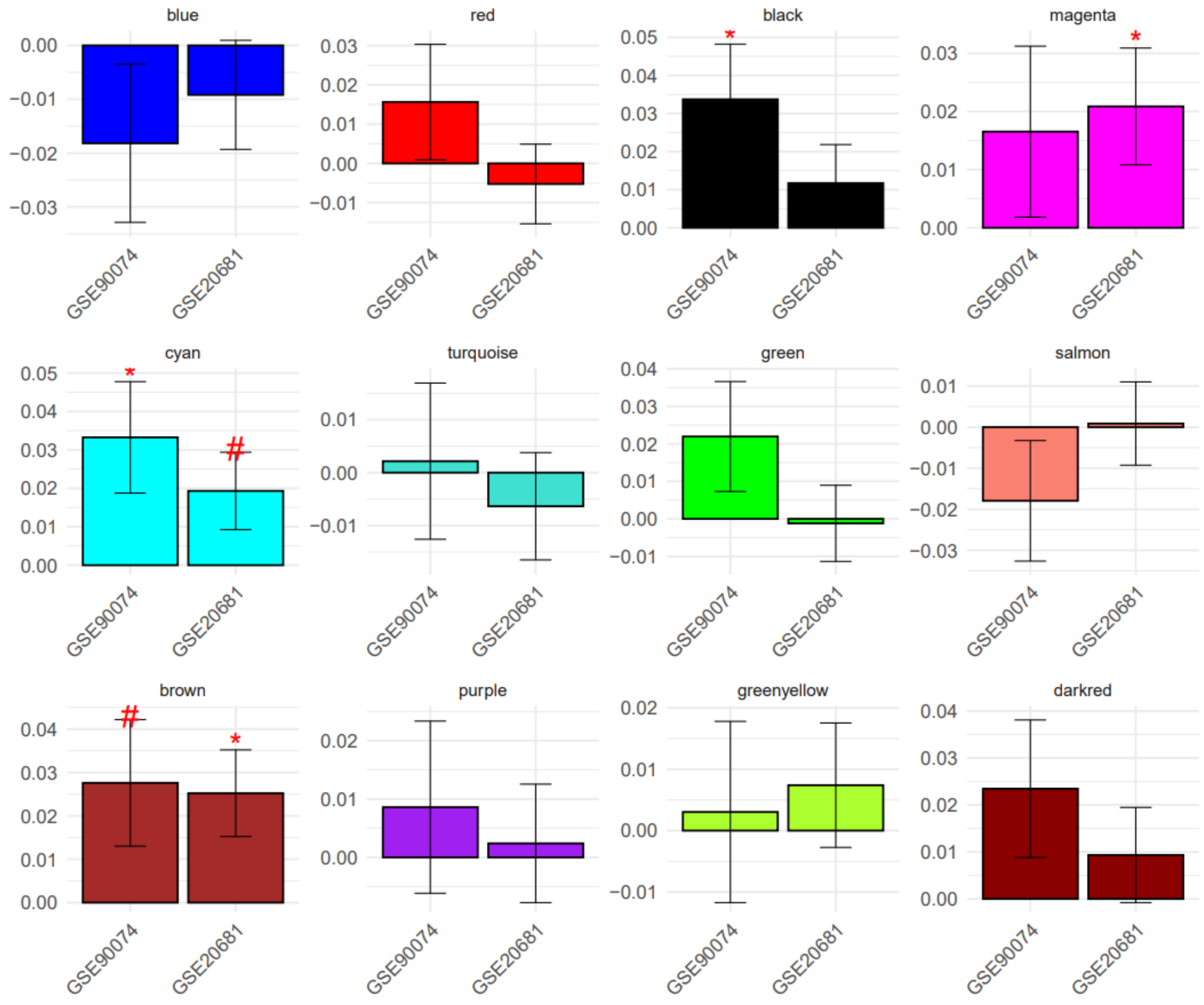

Supplementary Figure S6. Obstructive CAD-related modules selection based on the differential expression analysis (continued).

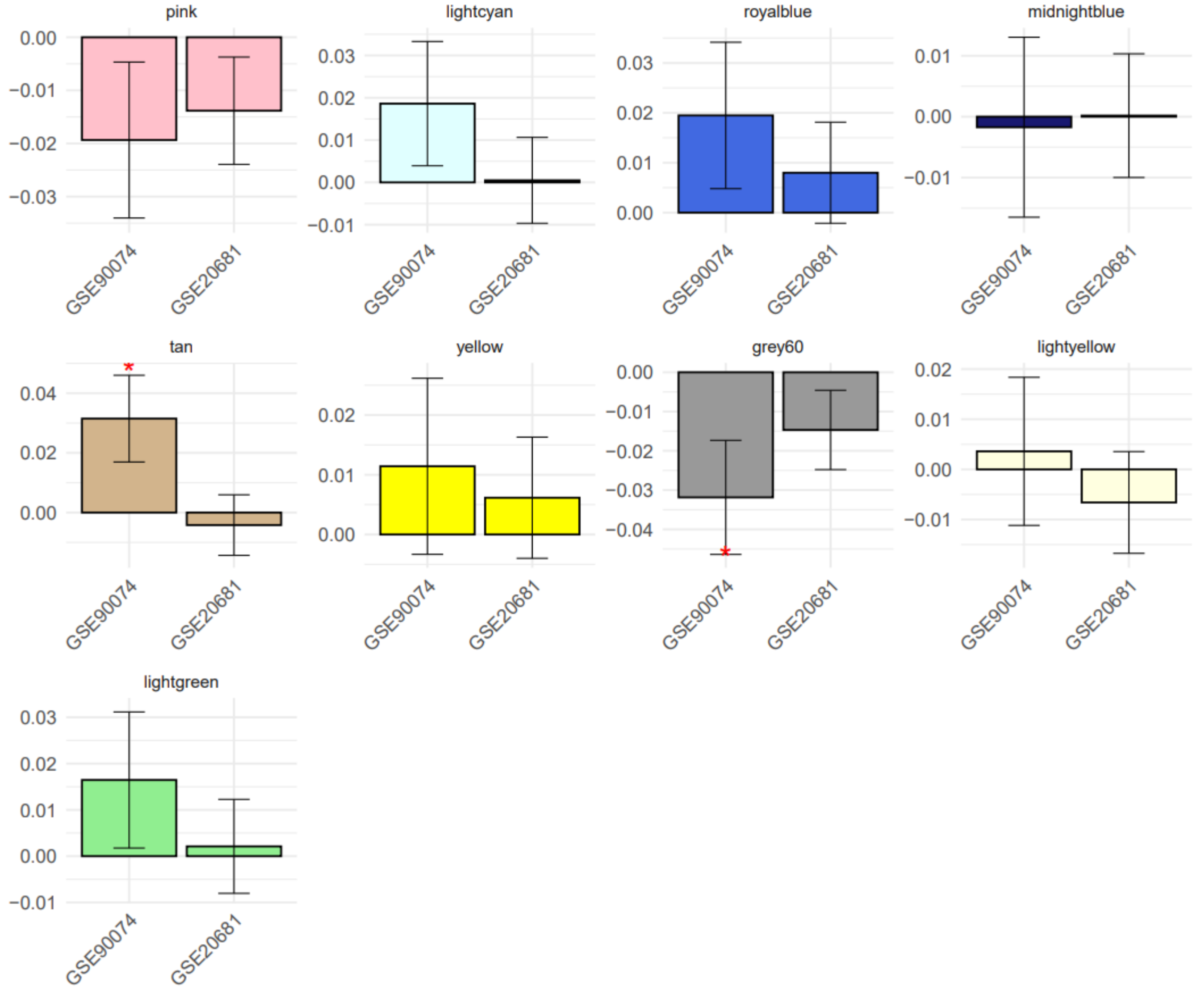

Supplementary Figure S7. ACS-related modules selection based on the differential expression analysis.

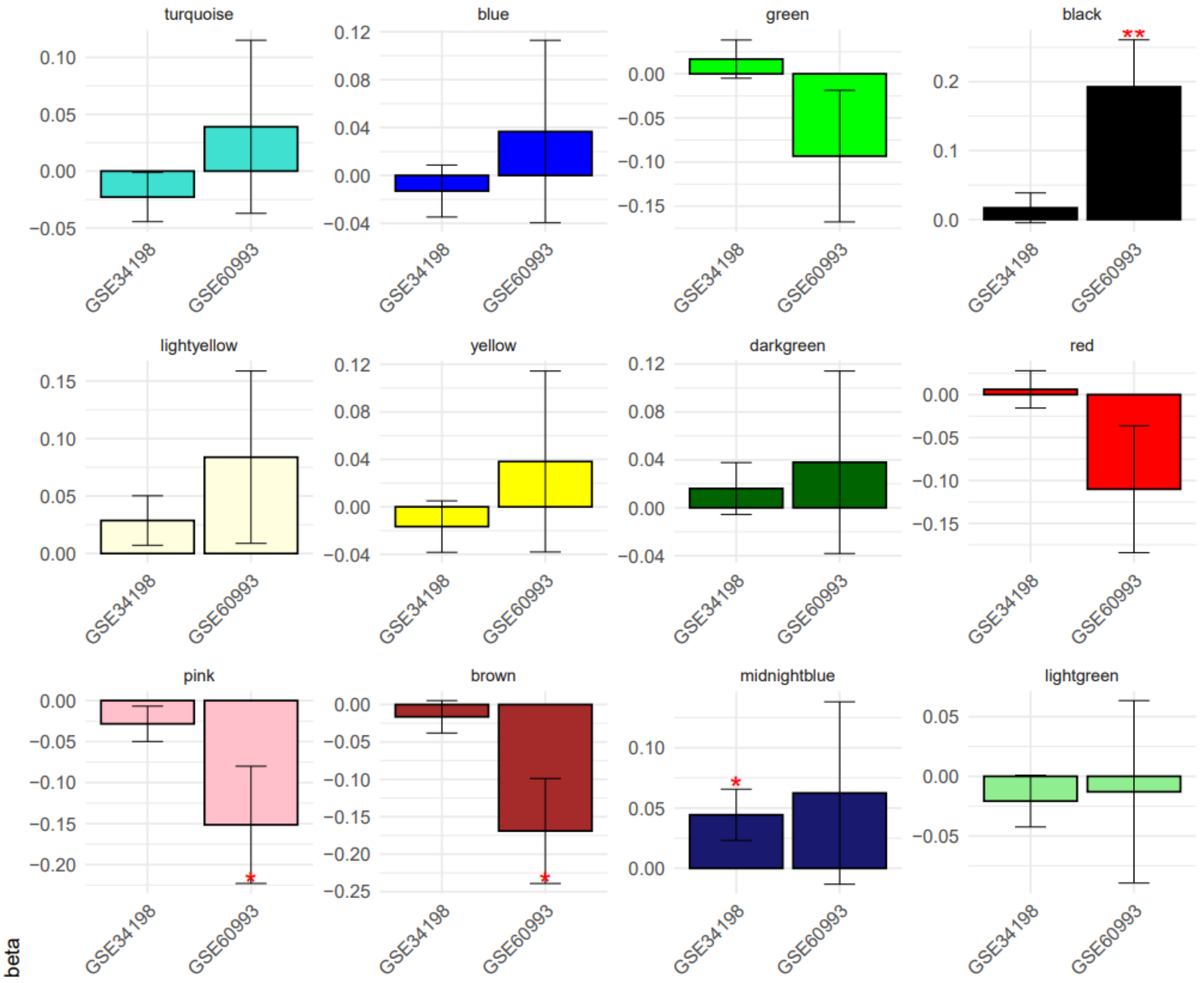

Supplementary Figure S7. ACS-related modules selection based on the differential expression analysis (continued).

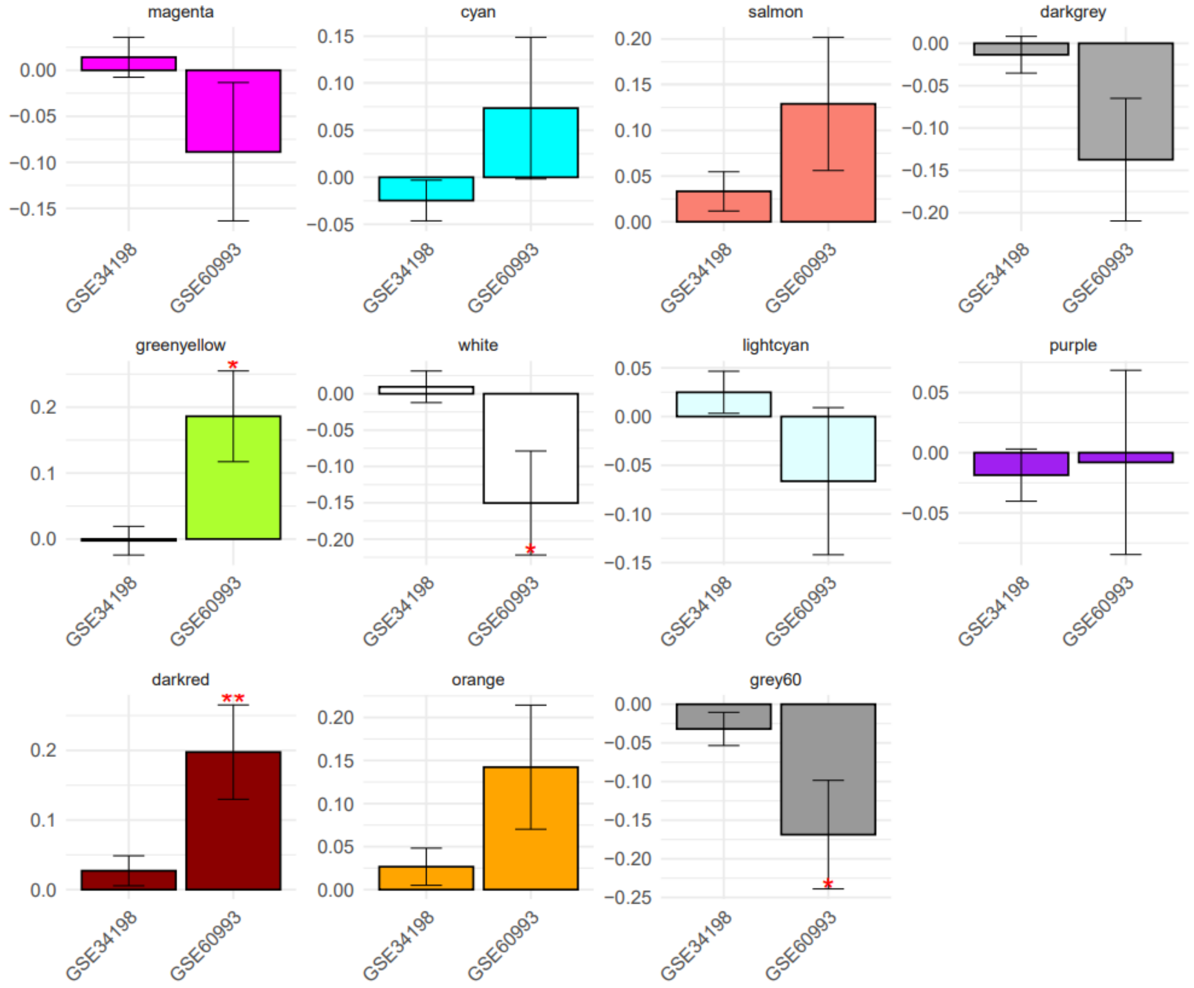

Supplementary Figure S8. Module selection based on the differential co-expression analysis.

(A)

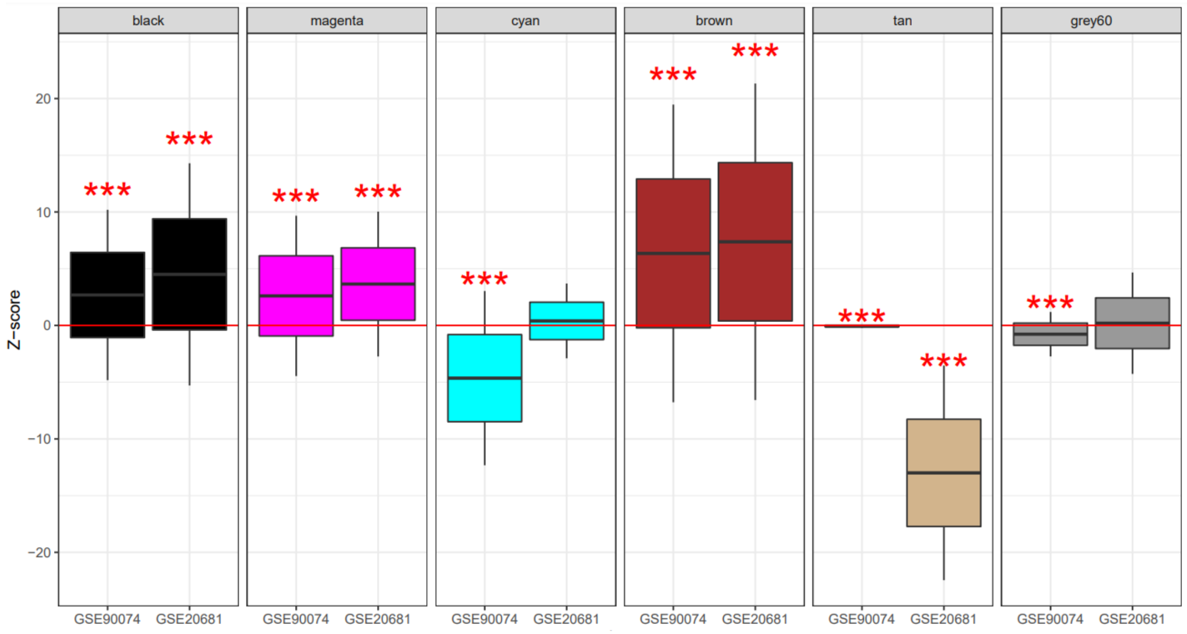

(B)

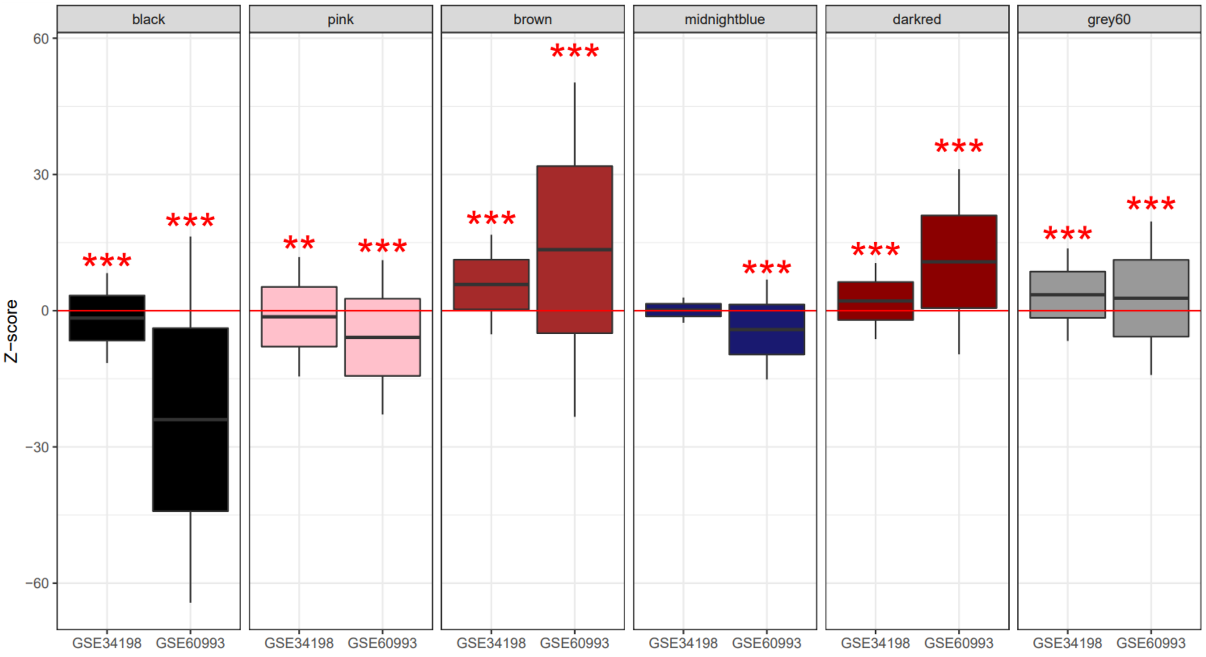

Supplementary Table S1. Summarization of human and mouse transcriptomic dataset.

Datasets	Study design	# of disease group	# of matched control	# of transcripts, probes, and probe-sets
GSE90074	Obstructive CAD vs. non-obstructive CAD	93	50	41,093
GSE20681	Obstructive CAD vs. non-obstructive CAD	99	99	45,015
GSE34198	ACS vs. non-ACS	45	48	48,701
GSE60993	ACS vs. non-ACS	26	7	48,803
GSE59867	ACS vs. non-ACS	111	46	33,297
GSE4648	ACS vs. Sham	36	24	12,488
GSE49937	SR-BI$^{-/-}$/apoE$^{-/-}$(dKO) vs. SR-BI$^{+/-}$/apoE$^{-/-}$(HET)	32	28	12,488
GSE153485	ACS vs. Sham	10	10	21,838
GSE775	ACS vs. Sham	18	18	12,488

Supplementary Table S2. Number of transcripts, probe, or probe-sets in three blood gene expression datasets according to pre-processing steps.

**Preprocessing Step**	**GSE90074**	**GSE20681**	**GSE34198**	**GSE60993**
**Original datasets**	41,093	45,015	48,701	48,803
**Removing transcripts, probes, or probe-sets without Entrez-ID**	30,936	32,696	30,541	35,962
**Removing 40% of transcripts, probes, or probe-sets based on low variance across samples**	18,561	19,617	18,324	21,577
**Gene (Entrez-ID)-based selection of transcripts**	12,598	12,867	17,823	16,322

Supplementary Table S3. Genes in turquoise and yellow modules

Black module
ABHD18, ABI1, ACTB, ACTG1, ADAM17, AFF1, AIDA, AKTIP, AMACR, ANKRD10, AP3S1, ARCN1, ARFGAP3, ARHGAP1, ARHGEF2, ARID4A, ARNT, ATF1, ATP6V1B2, ATP6V1G1, ATP8B5P, BAZ2A, BEX4, BICRAL, BRD2, BTG1, C18orf25, C1orf52, C1orf56, C2orf68, C7orf25, C9orf72, CALM2, CAP1, CAPZA1, CAPZA2, CARD8, CCDC28A, CCNG2, CDC42SE1, CDC73, CDS2, CEMIP2, CHMP1B, CHUK, CLK1, CLP1, CNBP, CNEP1R1, COG3, COIL, COTL1, CPOX, CRADD, CRK, CTCF, CTDSP2, CTSS, CUTC, CWC22, CWF19L1, CYB5B, CYTH1, DDX5, DDX59, DEGS1, DENND5A, DGCR2, DHRS7, DNAJA1, DNAJB6, DR1, DTX3L, DUSP11, EAF1, EDEM1, EDEM2, EIF1, ELOVL5, EPG5, ERI1, EXOC1, EXOC5, EYA3, F2RL1, F8A1, FAM120AOS, FAM91A1, FBXL12, FBXO3, FMR1, FOS, GADD45A, GET1, GID8, GLE1, GLUL, GMCL1, GNB1, GPBP1L1, HMGN2, HMGN2P46, HNRNPCL1, HNRNPLL, HSPA5, IDI1, IER2, ILKAP, INO80C, IST1, ITPR2, KANSL2, KBTBD2, KCTD18, KDELR2, KDM2A, KDM3A, KLF2, KMT5B, KRCC1, LAMP1, LASP1, LATS2, LOC643454, LRRC42, LYRM1, MAML1, MAPK9, MARCHF5, MARCHF7, MCL1, MED23, MED8, MFSD14A, MOB1A, MORF4L1, MR1, MRFAP1, MRFAP1L1, MTM1, MTMR12, MYCBP, NBPF1, NBPF14, NPTN, NSL1, NT5C2, OAZ1, OGT, PAFAH1B2, PAIP2, PAN3, PAPSS1, PCBP1, PDE4B, PDE6D, PELI2, PEX13, PICALM, PIK3CA, PLEKHB2, PPP1R10, PPP2CA, PPP4C, PPP4R2, PPP6R3, PRDM2, PRDM4, PRUNE1, PSMD5, RAB1A, RAB21, RAB5A, RAB8A, RAD23B, RALB, RALBP1, RANBP9, RAP1A, RAP2C, RARA, RBM33, RBMX2, RMI1, RNF122, RNF146, RNF20, RRAGC, RTF1, RUNX2, SAFB, SAMSN1, SARAF, SBNO1, SDHC, SEC22B, SELENOT, SETD3, SF3B1, SIAH1, SLC35A5, SLC35B3, SLC35F5, SLF2, SMAD2, SMAD4, SMARCA2, SMIM14, SNAP23, SPG21, SPIDR, SRF, SRGAP2C, STAG1, STAMBP, STAT5A, STK24, STYXL1, SUDS3, SUMO1, TAB2, TANK, TCF20, TLE3, TMBIM4, TMBIM6, TMED2, TMED7, TMEM165, TMEM248, TMEM50A, TNRC6B, TOP1, TOP1P2, TOR1A, TOR1AIP1, TUBGCP3, TVP23C, TXNDC12, TXNRD1, UBA3, UBE2G1, UBE2J1, UBR5, UHRF1BP1L, UPF2, VBP1, VEZF1, VMP1, VPS41, VPS4B, VTA1, WTAP, XRN2, YTHDF3, YWHAH, ZFP91, ZNF141, ZNF200, ZNF230, ZNF267, ZNF274, ZNF586, ZNF678, ZNF728, ZNF776, ZSCAN32
Magenta module
ABCA1, ACOX1, ACSL4, AGFG1, AGTPBP1, AHCTF1, AMD1, AMN1, ANP32A, ARG1, ARHGAP15, ARHGAP19, ARHGAP26, ARPC3, ATP6V1A, ATP6V1C1, AVIL, AZIN1, B3GNT5, BACH1, BASP1, BAZ2B, BEND7, BNIP2, C18orf32, CCDC153, CCPG1, CD302, CDH26, CDK14, CDKL5, CEP19, CFAP92, CHD7, CHMP2A, CHRNA10, COP1, CPD, CPEB4, CPPED1, CREB5, CREBBP, CRISPLD2, CXCR4, CYB5R4, CYP4F3, DACH1, DCP2, DCTN4, DHRS12, DHRSX, DRC1, DYNC1LI1, EGLN1, ENTPD1, ERGIC1, ERLIN1, ERO1A, EVI2A, F5, FAM120A, FAM126B, FAM209A, FAM8A1, FAR1, FBXL5, FBXO30, FBXO33, FBXO38, FCHO2, FGGY, FHIP2A, FNDC3B, GAB1, GALNT7, GCA, GDAP2, GK, GPAT3, H3C1, H3C11, H3C15, H3C4, HBP1, HCG27, HDAC4, HECW2, HHEX, HNRNPH2, HSD17B11, HSDL2, IFNAR1, IFNGR1, IFRD1, IKBIP, IL1RAP, INHBB, IP6K1, IQGAP1, IRAG2, IRS2, KATNBL1, KBTBD7, KCNE3, KDM5B, KIAA0232, KIAA0319, KIF13A, KIT, KLF5, KLHL2, KLHL8, LCOR, LIN7A, LMBRD1, LMNB1, LPCAT2, LRRC4, LRRK2, LXN, LY96, MAP2K6, MAP3K2, MMADHC, MSL3, MSL3P1, MSRB2, MTMR6, MVP, NFE2L2, NHS, NIN, NPEPPS, NQO2, NRDC, NSUN7, NUFIP2, NUP58, OPLAH, ORM1, ORM2, OSBPL1A, OSBPL8, PACSIN2, PARP8, PCMTD2, PDZD8, PELI1, PHLPP1, PIP4P2, PJA2, PLAGL1, PLXNC1, PPP2R2A, PRCP, PRKAR1A, PTBP3, PTGS2, PYGL, QKI, RAB32, RAB36, RAD21, RB1CC1, RBM47, RBP7, RBPJ, RCOR1, REPS2, RESF1, RGL3, RHOA, RICTOR, RILPL1, RIPOR2, RNASEL, RNF103, RNF111, RNF13, RPGR, RPS6KA5, RRM2B, RYBP, SEPTIN14, SERPINB1, SH3GLB1, SHOC2, SLC12A6, SLC16A3, SLC19A1, SLC22A15, SLC22A4, SLC26A8, SLC37A3, SLC40A1, SNX10, SNX18, SOS2, SP1, SPOPL, SRGN, ST3GAL6, ST6GALNAC3, STAM2, STK38L, STX10, STX3, SULT1B1, SYNJ1, TET2, THBD, TIMP2, TKT, TLE4, TLR1, TLR4, TLR6, TMEM33, TMEM59, TMEM71, TMEM88, TMLHE, TNNI2, TRIQK, TUT7, TXN, UBE2W, UBXN2B, UGGT1, USP10, VAMP3, VNN2, VNN3P, VPS8, WDFY3, WIPF2, YIPF4, YPEL5, ZBTB34, ZFAS1, ZFP36L1, ZFYVE16, ZMPSTE24, ZNF281, ZSWIM6
Brown module
AATK, ABCG1, ABHD2, ABHD3, ABHD5, ACSL1, ACSL3, ADAM10, ADAM8, ADAM9, ADM, AGO4, AKIRIN1, AKIRIN2, ANKRD13A, ANKS1A, ANO10, ANXA3, AOC3, APAF1, APBB1IP, APMAP, APPL2, AREL1, ARHGEF40, ARL8A, ARPC5, ASPH, ASPRV1, ATF6, ATG16L2, ATP11A, ATP11B, ATP6V0E1, ATXN1, AVL9, B2M, B4GALT1, B4GALT5, B9D2, BASP1-AS1, BCL2A1, BCL3, BCL6, BMX, BRI3, BTBD10, C11orf54, C16orf72, C1RL, C3orf62, C4orf3, CAB39, CACUL1, CAMKK2, CARD6, CARS2, CASC3, CASP4, CCDC71L, CCNJL, CD55, CD58, CD59, CD63, CDC123, CDKN2D, CEBPB, CEBPD, CHIC2, CHP1, CHST15, CHSY1, CKAP4, CKLF, CLIP1, CMTM6, CNIH4, CNTNAP3, CORO1C, CPQ, CR1, CSGALNACT2, CTBP2, CTBS, CUL4B, CWC25, CXCL1, CYSTM1, DAZAP2, DCUN1D1, DDIT3, DENND10, DHTKD1, DICER1, DOK3, DPH3, DPH3P1, DPY19L3, DRAM1, DUSP1, DUSP13, E2F3, ECHDC3, ELL, EPOR, ETS2, ETV6, EXOC6, F11R, FADD, FAR2, FAS, FBRS, FBXL13, FCAR, FGD4, FKBP5, FRAT2, FTH1, GABARAPL1, GABARAPL3, GALNT14, GMFG, GMPR2, GNAI3, GNG10, GNG5, GNS, GPER1, GPR160, GPR27, GRB10, GTF2I, GYG1, H2AZ1, H3-3A, H3-3B, H3-4, H3-5, H3C13, H3C2, H3C3, H3C8, HAL, HAUS4, HCLS1, HEBP2, HINT3, HK2, HMGB2, HSPA1A, HTATIP2, IFNGR2, IGF1R, IGF2R, IL13RA1, IL1R1, IL4R, IMPDH1, ING1, INPP5A, IRAK3, ITPRID2, JDP2, JPT1, JUNB, KCNE1, KCNJ2-AS1, KDM3B, KIF1B, KLF7, KLHL12, KLHL21, KPNB1, KREMEN1, KRT23, LAMP2, LAMTOR3, LAMTOR5, LAT2, LBR, LCP1, LIMK2, LINC01000, LPGAT1, LRRFIP2, LRRN1, MAEA, MAK, MAN2A2, MANSC1, MAP2K4, MAP3K3, MAP3K5, MAP4K4, MAPK1, MAPK14, MARCKS, MBOAT2, MCTP2, MEGF9, METTL9, MFN2, MFSD14B, MGRN1, MINDY1, MKNK2, MLX, MME, MMP9, MPZL1, MPZL3, MSL1, MSRA, MSRB3, MTARC1, MTFMT, MTHFS, MTMR3, MXD1, MYL12A, MYL12B, MYL6, MYLIP, NABP1, NACC2, NAMPT, NDUFB3, NEDD9, NFE4, NFIL3, NFKBIA, NIBAN1, NIPBL, NLRX1, NOL4L, NOP10, NOTCH2, NRBF2, NSMAF, NUAK2, NUDT5, NUMB, OAT, OSBPL2, OSER1, OSGIN2, OSTF1, OXSR1, PDK3, PHF21A, PHTF1, PIGX, PIM3, PITPNA, PLAUR, PLBD1, POLD3, PPFIA1, PPIAP51, PPP1R12B, PPP1R3B, PPP2R5A, PPP3CA, PPP4R1, PREX1, PRKDC, PRR13, PSEN1, PSMB3, PSMD4, PTP4A1, PTPRJ, PTTG1IP, QPCT, QSOX1, RAB11FIP1, RAB18, RAB27A, RAB2A, RAB31, RAB3D, RAB43, RAB5IF, RALGAPA2, RASSF2, RFLNB, RGS2, RILPL2, RIT1, RLIM, RNF130, RNF144B, RNF149, ROPN1L, RRAGD, RTN4, S100A6, S100P, SAT1, SCYL1, SDCBP, SERTAD3, SFT2D1, SIPA1L2, SIRPA, SKAP2, SLA, SLC12A9, SLC15A4, SLC16A5, SLC25A44, SLC2A14, SLC2A3, SLC31A2, SLC38A2, SLC43A2, SLC49A4, SLC6A6, SLC8A1, SLCO4C1, SMCHD1, SNX13, SNX27, SOCS3, SOD2, SORL1, SORT1, SPAG9, SQOR, SRPK1, SSH3, ST6GALNAC2, STEAP4, STK17B, STK40, STX11, STX6, STXBP5, SUSD6, SVIL, TADA3, TALDO1, TBC1D14, TDP2, TECPR2, TESMIN, TGFA, THEMIS2, TM9SF2, TMCO3, TMEM127, TMEM167A, TMEM184B, TMEM185B, TMEM30A, TMEM43, TMEM45B, TMEM65, TMX4, TP53INP2, TPD52L2, TPST1, TRIM8, TSEN34, TSHZ3, TUBA4A, U2AF1, UBALD2, UBE2R2, UBL5, UBR2, UIMC1, ULK1, UPF1, USB1, USP3, USP6, USP9X, VAPA, VCPKMT, VIM, WAC, WIPI1, WLS, WWC3, YIPF1, ZDHHC3, ZNF438, ZNF516, ZNF746, ZXDC

Supplementary Table S4. Genes in turquoise and yellow modules

Pink module
ABCF1, ABHD10, ABL1, ACO2, ACOT2, ACOT7, ADAM15, ADSL, AIMP2, AK2, AKR7A2, ALG3, ALKBH4, ALKBH6, ALKBH7, ANKMY1, ANKS3, AP4B1, AQR, ARFGAP2, ARHGAP17, ARHGEF19, ARL2, ARRDC1-AS1, ASPSCR1, ATF5, ATP5F1A, B4GALT7, BLOC1S4, BMS1, BOP1, BORCS6, BPHL, BRD9, BSCL2, BTBD11, C11orf24, C16orf91, C19orf12, C19orf44, C19orf48, C1orf50, C20orf27, C2CD2, C4orf48, CALM3, CAPS, CBR1, CBX6, CBY1, CCDC106, CCDC136, CCDC25, CCDC92, CCT2, CCT7, CD1C, CD320, CD7, CDC37, CDK9, CENPB, CENPX, CEP131, CES2, CLCN7, CLSTN1, CNDP2, CNOT11, COMT, COX4I1, CPSF1, CPSF3, CPSF4, CTC1, CTNS, CXXC1, DCXR, DDX19A, DDX51, DENND4C, DGCR6, DGCR6L, DGUOK, DHRS1, DIMT1, DNAH1, DNAJC17, DNAJC8, DPAGT1, E2F6, E4F1, EBP, ECI1, EDC4, EEF1D, EIF2B1, EIF2B4, EIF2B5, ELP6, EML3, ENO3, ERGIC3, ERI3, ETFB, EVI5L, EVL, EZR, FAAP100, FAAP20, FAM110A, FAM50A, FANCE, FASN, FBXL6, FDXR, FIBP, FKBP11, FKBP4, FLNB, FN3KRP, GALT, GATB, GATD1, GMDS, GNA11, GNL1, GPN2, GPS1, GTPBP6, HADHA, HARS1, HAUS7, HDAC1, HDAC11, HGH1, HIRIP3, HLA-DMA, HMCES, HNRNPC, HPS6, IL12RB1, ILKAP, JMJD7-PLA2G4B, JSRP1, KDM1A, KHSRP, KLHDC3, KLHL22, KPNA6, KRBA1, KRTCAP2, KYAT1, LAT, LHPP, LINC01089, LMNA, LOC653303, LRRC23, LRRC41, LRRC45, LTO1, MADD, MAGED2, MAN1B1, MED16, MED22, METTL16, MFGE8, MFSD10, MFSD3, MPDU1, MPND, MRGBP, MRM3, MRPL37, MRPS16, MRPS18A, MRRF, MTFP1, MVB12A, MZF1, NACA, NAXE, NDRG2, NDUFS3, NDUFS7, NEK8, NFKBIE, NICN1, NMRAL1, NMT1, NOD1, NOLC1, NOP16, NOP2, NPRL2, NR2C2AP, NSDHL, NSMCE1, NSUN5P2, NT5C, NUDT1, NUP37, NUP42, NUP85, NVL, OSBP, OSGEP, PACSIN1, PAOX, PARP6, PCCB, PDCL3, PDIA4, PES1, PEX10, PEX11B, PEX16, PEX26, PGRMC2, PHB1, PHGDH, PHRF1, PI4KA, PIGU, PIH1D1, PLCH2, PLXND1, POLD2, POLE, POLG, POU2F2, PPIE, PPP1R35, PPP1R3E, PRMT7, PRPF19, PRR3, PRRT2, PSMB10, PSMC3, PSMD3, PSMD8, PTBP1, PUF60, PUS1, QARS1, R3HCC1, RABGGTA, RBFA, RBM4B, RCC2, REXO4, RHOC, RIPOR1, RITA1, RNF113A, RNF126, RP9, RPL32, RPS2P8, RRP36, RRP8, RUSC1, RUVBL1, SARS1, SAYSD1, SCARB1, SCMH1, SCNN1D, SCRIB, SDHAF1, SDR39U1, SERPINB6, SF3A3, SF3B2, SGF29, SH2B1, SHFL, SIL1, SLC25A10, SLC25A19, SLC25A26, SLC25A3, SLC27A3, SLC2A4RG, SLC2A8, SLC41A3, SLX9, SMARCA4, SNAPC2, SNAPC4, SNHG11, SNRPB, SNU13, SNX5, SPAG7, SPATA20, SPG7, SPHK2, SPNS1, SPOUT1, SRRM1, SSU72, STK36, TACO1, TAPBPL, TARBP2, TCF25, TDP1, TEDC1, TELO2, TEPSIN, TEX261, THEM6, TICAM1, TIGD5, TIMM22, TMED3, TMEM121, TMEM175, TMEM203, TMEM205, TNFRSF14, TNPO2, TOMM34, TP53I13, TRABD, TRAF3IP2, TRAPPC4, TRIB3, TRMT1, TSPAN17, TSPAN18, TSPAN31, TSPAN32, TSSC4, TTC7A, TUBG1, TUBG2, TXLNA, TXNRD2, UBA7, UROS, USE1, UTP14A, VARS2, VPS16, WDR18, WDR70, WDR74, WDR83OS, WDR97, WRAP53, XBP1, YIF1A, YJU2B, ZBTB25, ZBTB3, ZDHHC16, ZNF142, ZNF212, ZNF296, ZNF317, ZNF34, ZNF358, ZNF511, ZNF622, ZNF777
Darkred module
ABHD5, ACOX1, ACSL1, ACTN1, ADM, AGO4, AGTPBP1, ALOX5AP, ANKS1A, ANTXR2, ANXA3, APMAP, AQP9, ARG1, ARHGEF40, ARID3A, ATP11B, ATXN1, AVIL, B4GALT5, BASP1, BCL6, BEST1, BMX, BRD8, BST1, BTBD10, C16orf72, C1RL, C3orf62, CAB39, CAMK2G, CBL, CCNJL, CCPG1, CD59, CEBPB, CHIC2, CHRNA10, CHST15, CLEC4D, CLEC4E, COP1, CPD, CREB5, CRISPLD2, CXCR2, CYRIA, DAPK2, DEF8, DNAJC25-GNG10, DNAJC3, DNTTIP1, DOCK5, DYNLT1, ECHDC3, EGLN1, ELF2, ELL, ERGIC1, ETS2, EVI2B, EXOC6, FADD, FAR2, FBXL13, FBXO38, FCGR3B, FHIP2A, FKBP5, FOS, FPR2, FRAT2, GAB2, GABARAPL1, GAS7, GBE1, GK, GPR141, H2BC4, H2BC6, HAL, HAUS4, HCG27, HECW2, HSDL2, HSPBAP1, HTATIP2, ICAM3, IFNAR1, IFNGR1, IFNGR2, IGF1R, IKBIP, IL13RA1, IL18R1, IL1R2, IRAK3, IRS2, ITPRIP, JPT1, KBTBD2, KCNJ15, KIAA0040, KIAA0232, KIAA0319, KIF1B, KLHL8, LAMP2, LAT2, LBR, LILRA2, LIN7A, LINC00921, LINC01191, LINC02649, LITAF, LMNB1, LPAR2, LPGAT1, LRRN1, LSMEM1, LY96, MAK, MAN2A2, MAP4K4, MAPK1, MBOAT1, MCEMP1, MGAM, MME, MMP25, MTARC1, MTHFS, MXD1, MYBPC3, NAMPT, NCF2, NDEL1, NEDD9, NFIL3, NIBAN1, NLRP12, NME8, NRBF2, NSMAF, NSUN7, ORM1, OSBPL1A, OSBPL2, OSER1, OSGIN2, OSM, P2RY13, PACSIN2, PADI4, PANX2, PDK3, PDZD8, PELI2, PFKFB3, PHF21A, PHTF1, PISD, PLBD1, PLXDC2, PPFIA1, PPP1R15A, PPP1R3D, PPP4R1, PROK2, PTEN, PXK, PXYLP1, PYGL, QPCT, RAB2A, RAB31, RAB7A, RAF1, RALB, RASSF3, REM2, RFX2, RGL2, RILPL1, RIT1, RNF130, RNF144B, RNF149, RTN3, RUBCNL, S100A12, SAT1, SERPINB1, SIPA1L2, SIRPA, SKAP2, SLC12A6, SLC22A15, SLC22A4, SLC25A44, SLC26A8, SLC2A3, SLC31A2, SLC36A1, SLC37A3, SLC45A4, SLC49A4, SLC9A8, SPAG9, SPINK8, SRGN, SRPK1, SSH2, ST6GALNAC2, STK3, STX11, STX3, STX6, STXBP5, SULT1B1, SVIL, TBC1D14, TGFA, TLR2, TLR4, TLR5, TLR8, TM6SF1, TMCC3, TMCO3, TMEM185B, TMLHE, TMX4, TRIB1, TRIM25, TSHZ3, TXN, UBE2B, UBR2, UHRF1BP1L, USP10, USP4, VAV3, VCPKMT, VMP1, VNN1, VNN2, VNN3P, VPS8, ZBTB34, ZFYVE16, ZNF20, ZNF438, ZNF516, ZNF746
